# Supplementary figures and images for: The Nestin neural enhancer is essential for normal levels of endogenous Nestin in neuroprogenitors but is not required for embryo development
Source: PLoS One. 2021 Nov 5;16(11):e0258538. doi: 10.1371/journal.pone.0258538 (PMC8570527; doi:10.1371/journal.pone.0258538)

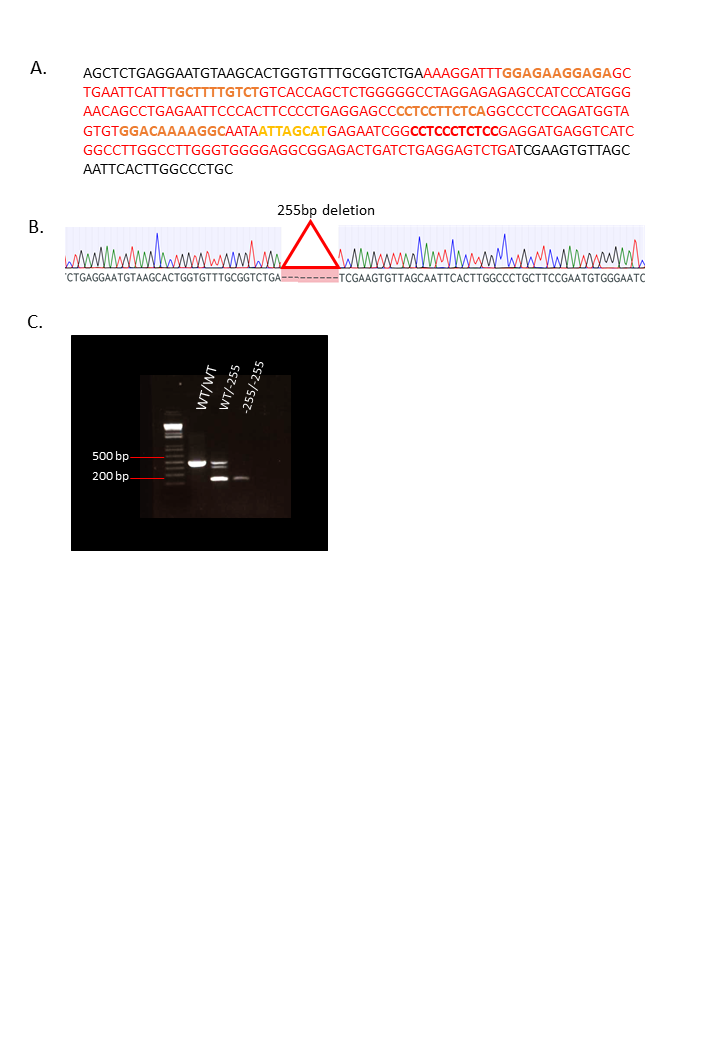

Supplement: S1 Fig — The mutation generated via CRISPR removed 255bp of DNA within intron 2 of Nestin. (A) The deletion in shown in red text, with SOX sites in bold and POU sites in yellow. (B) Chromatogram of the Nestin deletion showing the position of the 255 deletion. (C) Genotyping gel showing band sizes of the WT/WT, WT/-255 and -255/-255 samples. Note heteroduplex band in the WT/-255. (TIF) [file pone.0258538.s001.TIF]

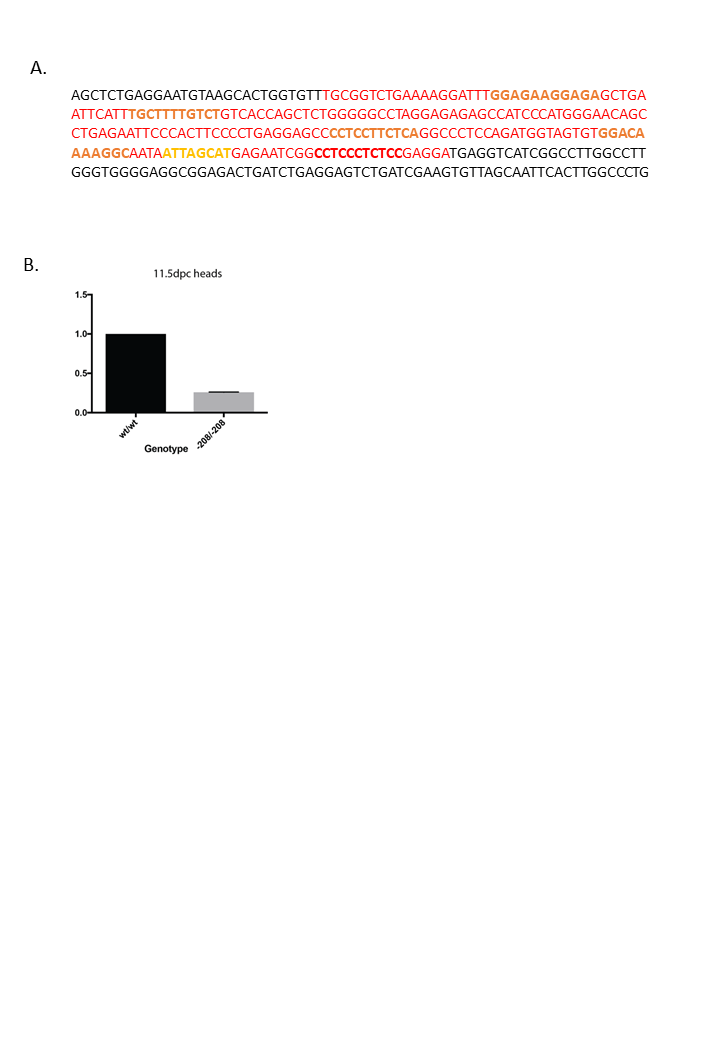

Supplement: S2 Fig — The -208 Nestin enhancer deletion line shows a reduction in Nes expression in 11.5 dpc embryonic heads similar to that of the -255 line (n = 2 embryos). (TIF) [file pone.0258538.s002.TIF]

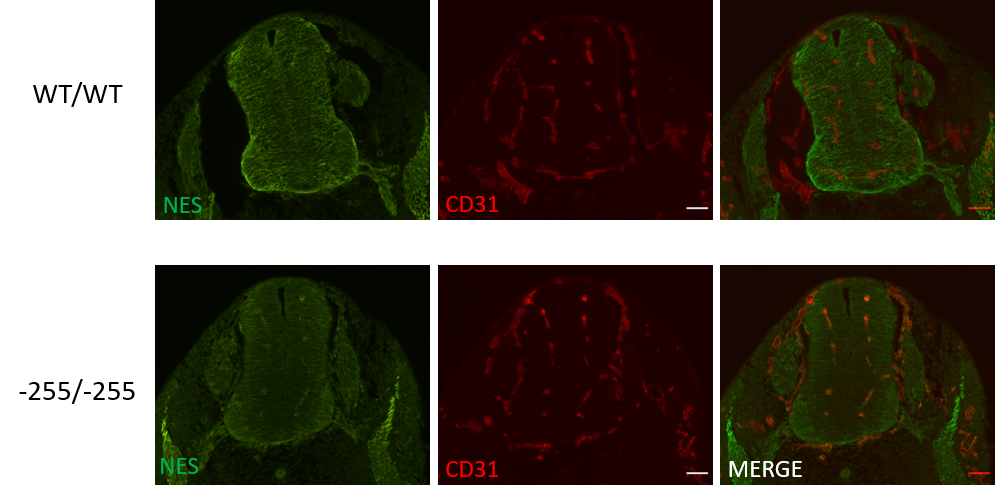

Supplement: S3 Fig — Nestin and CD31 expression within a 10.5dpc neural tube section. Within the WT sample no overlap is seen between CD31 and NES, while the -255/-255 sample shows co-localisation between the two proteins. (PNG) [file pone.0258538.s003.png]

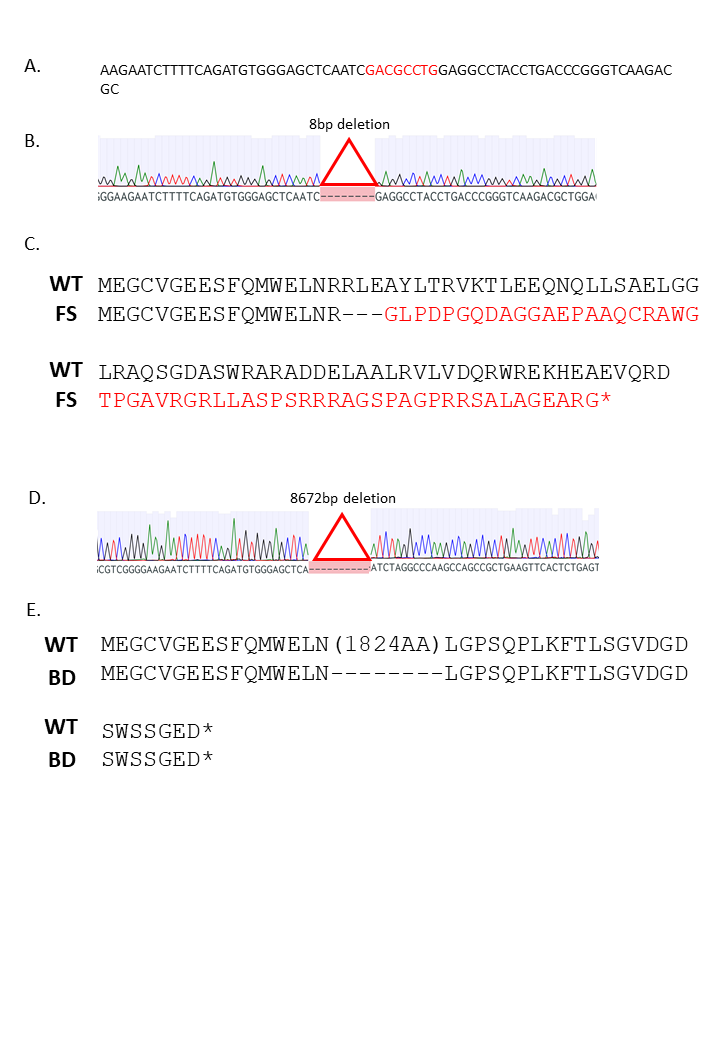

Supplement: S4 Fig — The Nestin ‘Big Deletion’ and ‘Frameshift Mutations’ generated by CRISPR. A. The FS mutation comprises an 8 bp deletion shown in red. B. The chromatogram file of the mutation. C. The amino acid sequence of the mutations generated by the FS mutation. D. The chromatogram file of the ‘Big Deletion’ incorporates an 8.6kb deletion that has been minimised for visualisation. E. The amino acid sequence of the BD mutation. The large 1824AA sequence within the WT sequence is not shown. (TIF) [file pone.0258538.s004.TIF]
